# Text cryptography and steganography based on RNA sequences generation, true random number generators, and codebook method

**DOI:** 10.1371/journal.pone.0338700

**Published:** 2025-12-09

**Authors:** Safwat Hamad, Omar Fitian Rashid, Hind M. Al-Dabbas

**Affiliations:** 1 Department of Mathematics, College of Science, University of Anbar, Ramadi, Iraq; 2 Department of Geology, College of Science, University of Baghdad, Baghdad, Iraq; 3 Department of Computer Science, College of Education for Pure Science/Ibn Al-Haitham, University of Baghdad, Baghdad, Iraq; University of Anbar, IRAQ

## Abstract

Classic security methods are becoming vulnerable because of technology advancements. The process of combining cryptography and steganography led to overcoming this, where this combination will save the information from potential attackers. This paper proposed a new text security method by combining the cryptography technique and steganography technique, where cryptography is used to encode the message, while steganography is used to hide the ciphertext within an image. The proposed cryptography system has five steps. The first is done by building an RNA sequences generation table to convert the message to RNA sequences, the next step is done by creating a table to convert RNA sequences to decimal numbers. In the third step, the achieved decimal values are converted to binary numbers; after that, used True Random Number Generators method generates the key, and finally, an XOR operation is applied between achieved binary numbers and the generated key. The results achieved by the XOR operation are considered as the ciphertext. Two steps to the proposed steganography method. The first step is done by building a codebook table to select the part of the pixel to store the bit inside it, where the selection part can be either red, green, or blue, and then used a least significant bit to store the bit with no effects on the image. The proposed method presents an efficient solution to secure data, and this method can be applied in different fields, such as information security and privacy protection.

## 1. Introduction

The process of data transmission has become critical due to the increase of the device’s computational power, leading to more complex attacks; therefore, traditional cryptographic systems can detect a few of these threats. DNA/RNA cryptography uses the concept of the transmission of hereditary information from one generation to another [1]. Where RNA has high storage capacity, fast processing, and high computation capacity, and is more secure than other cryptography algorithms. One of the advantages of using RNA cryptography is can be applied to both symmetric and asymmetric cryptography [2]. RNA includes the genetic information of beings, and it consists of four bases called Adenine (A), Cytosine (C), Guanine (G) and Uracil (U) [3].

Cryptography methods are proposed in different publication researches which are used to secure data to be transmitted over the internet. A new DNA cryptography method is proposed [4], where the proposed method is done by using a DNA encoding table to encode characters into DNA sequences with the using of mathematical series. A novel DNA cryptography method is generated by Beggas & Lounici [5]; this method generates random keys for One Time Pad cryptosystems, where the proposed method uses the structure of the DNA, some mathematical operations and DNA mechanism. A novel cryptosystem method is built by using DNA cryptography and finite automata theory; this method starts by generating a key with a length of 256 bits and uses it for the encryption process, then using the mealy machine method to encode the DNA [6]. Monika and Upadhyaya [7] proposed a new secure communication idea to provide a secure network channel by using DNA cryptography and a secure socket layer. A novel cryptosystem is suggested [8], where this method combines both DNA cryptography and DNA steganography for the cloud; this is done by using a long key to encrypt the information and then hide the encrypted information within an image. A new cryptography idea is proposed [9] based on DNA Cryptography where the proposed method starts by converting the message to ASCII values and then converting these values to binary format; after that, it converts binary to DNA sequences then convert these sequences to RNA sequences, and the last step is converting RNA sequences to the amino acid. A new encryption system is suggested by using both DES and AES [10], where the main idea is done by merges DES keys with AES keys to get 128 bits as a root key. Chiadighikaobi & Katuk [11] reviewed different method for g lightweight cryptography in IoT environment to bridge the gap in the different studies by conducting a systematic scoping study.

On the other hand, the steganography technique is used in various research to hide information within text, images, or video. A new image steganography method is proposed [12] by using adversarial learning theories, where a saliency map is used to add a value to each pixel based on its influence, which gives each pixel its importance in deceiving the steganalysis. A new method to secure transmission was proposed [13] based on two techniques which are compression and steganography, where the compression sensing method is used to compress multiple secret images, then apply encrypted to an image and hide it within the multiple images. Darani et al. [14] proposed a new image steganography method to insert a grayscale secret image into an RGB colour cover image by using a chaos map and 3D shift function. A new method to enhance the steganography method is proposed [15] based on modifying the LSB or 2nd LSB of stego-images; this method generates content-adaptive adversarial perturbations, then uses lossless run-length encoding method for data compression to store it within the image. Yang et al. [16] proposed a novel image steganography method by segmenting the threshold to process the image. They divided the image into two regions, which are foreground and background regions, to improve the embedding capacity. A novel security method is built by using both cryptography and steganography [17]; this method encodes the secret image based on DNA encoding and chaotic maps method, then hides the data within the image. Sharma et al.[18] proposed a new steganography method based on content-adaptive adversarial perturbation, where this method is done by creating a hybrid texture descriptor to describe texture regions and then segmenting the image into different parts by using simple linear iterative clustering. Investigated a new method to transform audio samples to spots on the curve [19] and also change the sample form, which makes the suggested method difficult to guess the curve points by an intruder. A new image steganography system is proposed using Discrete Shearlet Transform algorithm and secret sharing, where the proposed system have been obtained better hiding process and high embedding capacity [20]. Despite there are many efforts that have integrated DNA/RNA cryptography and steganography, most currently used techniques are based on deterministic or fixed key-generation algorithms or do not offer a dynamic embedding algorithm that maintains the quality of an image and is resistant to statistical detection. Also, very little literature combines the use of true random number generation with RNA-based encoding in a single pipeline of text-security. Therefore, this study fills this gap by suggesting a cryptography-steganography hybrid model based on the RNA sequence production, TRNG-based keying, and LSB embedding framework based on codebook.

The rest of this paper is structured in the following manner. Section 2 contains the materials and methods, which outline the proposed RNA-based cryptography and the steganography process which is done by the codebook. Section 3 presents the experimental findings and performance analysis of the two stages cryptography and steganography. Section 4 concludes the paper and provides possible future research on how the proposed security framework can be improved.

## 2. Materials and methods

**Fig 1:**
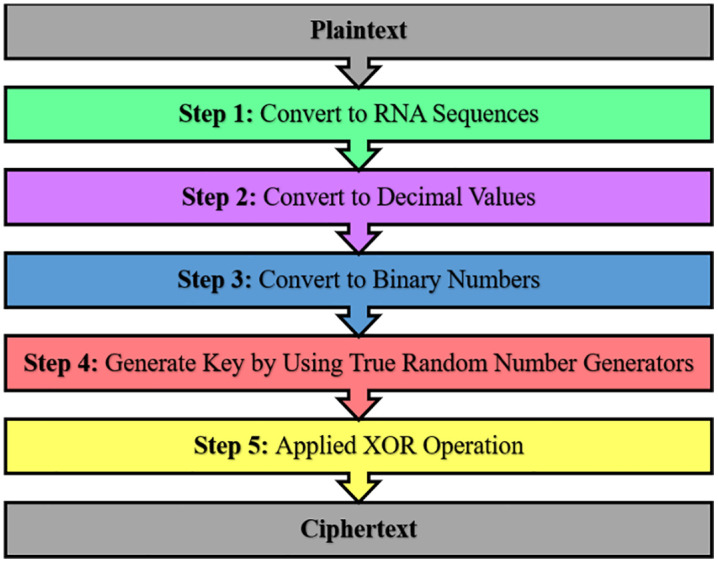
Proposed cryptography method steps.

The proposed method is applied to secure text transmission over the internet, where the proposed method has two phases. These phases are text cryptography and steganography. In the first phase, Cryptography is utilized to encode messages (plaintext) by using five steps. The proposed cryptography steps are shown in Fig 1.

The first phase of the proposed method is cryptography, where the main idea of this phase is to encode the plaintext. This method is done by building an RNA sequence generating table, where the table contains all possible characters that are used in plaintext. In the proposed method, the input can be a character, number, or special characters. The total possible value of these characters is equal to 72 characters; therefore, four random RNA characters can handle and represent all these values. This table is generated randomly, where each character has its unique RNA sequences, and this table is generated randomly every time a new message is sent. An example of RNA sequence generation for one time is listed in Table 1.

**Table 1:** RNA sequences generation.

No.	Character	RNA Sequences	No.	Character	RNA Sequences
1	A	GGGC	37	k	CAAC
2	B	CUAU	38	l	UCAC
3	C	UGAC	39	m	AUGU
4	D	UUCU	40	n	UCGA
5	E	UAUC	41	o	GCUA
6	F	GUAG	42	p	UGCA
7	G	CCGG	43	q	CAUA
8	H	CCUU	44	r	ACGU
9	I	GUUA	45	s	GCUU
10	J	AUGA	46	t	GGUC
11	K	GAAU	47	u	CCGA
12	L	CACC	48	v	CGUG
13	M	CUUC	49	w	CGAG
14	N	CAAG	50	x	CGUC
15	O	CCGC	51	y	AGCU
16	P	GAAG	52	z	CAGG
17	Q	ACUC	53	0	GUAU
18	R	UCCU	54	1	AUAA
19	S	GGCA	55	2	UCCA
20	T	GGUU	56	3	AAAG
21	U	AGUC	57	4	GGAC
22	V	CCCA	58	5	GUCU
23	W	AUAC	59	6	GAGG
24	X	CCAC	60	7	ACCC
25	Y	CGGA	61	8	UUGC
26	Z	UGGC	62	9	GCCC
27	a	AUCU	63	(	UAGC
28	b	GUAA	64	)	AAAC
29	c	AACA	65	@	UGCU
30	d	AGGG	66	&	GAGU
31	e	UAAG	67	$	GUGC
32	f	CCAG	68	%	GAUG
33	g	UGAU	69	?	UAGU
34	h	CAGA	70	!	UCUU
35	i	UACC	71	=	GUUC
36	j	UUCG	72	Space	AAAA

After completing the first step, where the plaintext is converted to RNA sequences, the next step is done by generating a random decimal converting table. Where the total possible number of four RNA sequences is equal to 256 values. Therefore, a decimal conversion table is built where each four RNA characters is converted randomly to a decimal value. The converting RNA sequences to decimal values table is presented in Table 2.

**Table 2:** RNA Sequences to Decimal Converting.

Decimal Value	RNA Sequences	Decimal Value	RNA Sequences	Decimal Value	RNA Sequences	Decimal Value	RNA Sequences
1	GGGC	65	UGCU	129	CGGG	193	UUUC
2	CUAU	66	GAGU	130	CCCU	194	CUCC
3	UGAC	67	GUGC	131	ACAG	195	GGUA
4	UUCU	68	GAUG	132	UCCC	196	GAGA
5	UAUC	69	UAGU	133	AUGG	197	CCUG
6	GUAG	70	UCUU	134	GAGC	198	CUGA
7	CCGG	71	GUUC	135	AACG	199	AGAU
8	CCUU	72	AUUG	136	AAAA	200	UGAG
9	GUUA	73	CAAA	137	GGGG	201	UGCC
10	AUGA	74	GCAC	138	CUAG	202	UACA
11	GAAU	75	GUCA	139	UAUU	203	AAGA
12	CACC	76	ACAU	140	AGGU	204	AGAA
13	CUUC	77	GCCU	141	CCCC	205	AUCC
14	CAAG	78	CAGU	142	AGGC	206	GGCC
15	CCGC	79	CGCU	143	UAGG	207	UUGU
16	GAAG	80	AGUA	144	CUGC	208	CAUC
17	ACUC	81	UAUG	145	CUCA	209	AGAG
18	UCCU	82	GUGA	146	GCGC	210	CCGU
19	GGCA	83	UACU	147	CAGC	211	UUAU
20	GGUU	84	ACCG	148	GUCG	212	CGCC
21	AGUC	85	CGAA	149	AUCA	213	AGCC
22	CCCA	86	AUUC	150	AGUG	214	ACUG
23	AUAC	87	ACCA	151	UUGA	215	GUGU
24	CCAC	88	GAUC	152	CCAU	216	CGUA
25	CGGA	89	ACCU	153	ACGG	217	AAGG
26	UGGC	90	UUAA	154	UUCA	218	CAUU
27	AUCU	91	UUGG	155	CUGG	219	UCUG
28	GUAA	92	UUCC	156	AUAU	220	AGCA
29	AACA	93	ACGC	157	GCAG	221	AUUA
30	AGGG	94	GCGA	158	GCGU	222	AACU
31	UAAG	95	GAUU	159	GUAC	223	UUUG
32	CCAG	96	UCAG	160	UCAU	224	UCGU
33	UGAU	97	ACGA	161	AAUU	225	AAGU
34	CAGA	98	CCUC	162	GCAA	226	CUUA
35	UACC	99	GGCU	163	CCCG	227	GCAU
36	UUCG	100	GGAG	164	CAUG	228	UGCG
37	CAAC	101	GCUC	165	CUUU	229	CCAA
38	UCAC	102	GUCC	166	UGUU	230	GUGG
39	AUGU	103	ACUU	167	GACC	231	CGUU
40	UCGA	104	GGCG	168	UUUU	232	CGGC
41	GCUA	105	UUAG	169	AAGC	233	GGGU
42	UGCA	106	CACG	170	CUCU	234	GACG
43	CAUA	107	GGGA	171	CUUG	235	UGGG
44	ACGU	108	ACAA	172	CUAA	236	AAUC
45	GCUU	109	CGGU	173	CGAU	237	AUUU
46	GGUC	110	ACAC	174	GAAC	238	AGAC
47	CCGA	111	GAAA	175	CGAC	239	CACU
48	CGUG	112	AGUU	176	UGUC	240	GCUG
49	CGAG	113	GCCG	177	CUAC	241	AGCG
50	CGUC	114	GCCA	178	UAUA	242	AUAG
51	AGCU	115	UAAU	179	AAUG	243	AUGC
52	CAGG	116	UAAA	180	UAAC	244	GCGG
53	GUAU	117	UCCG	181	ACUA	245	GGUG
54	AUAA	118	UGUA	182	CAAU	246	UUAC
55	UCCA	119	CGCA	183	AUCG	247	UGUG
56	AAAG	120	GGAA	184	AAUA	248	AACC
57	GGAC	121	CGCG	185	GACU	249	CUCG
58	GUCU	122	GACA	186	UACG	250	UUUA
59	GAGG	123	UCGC	187	UCUC	251	CACA
60	ACCC	124	UCUA	188	UGAA	252	UCAA
61	UUGC	125	CUGU	189	GUUG	253	UAGA
62	GCCC	126	UGGA	190	CCUA	254	AAAU
63	UAGC	127	UCGG	191	GGAU	255	GAUA
64	AAAC	128	UGGU	192	GUUU	256	AGGA

**Fig 2:**
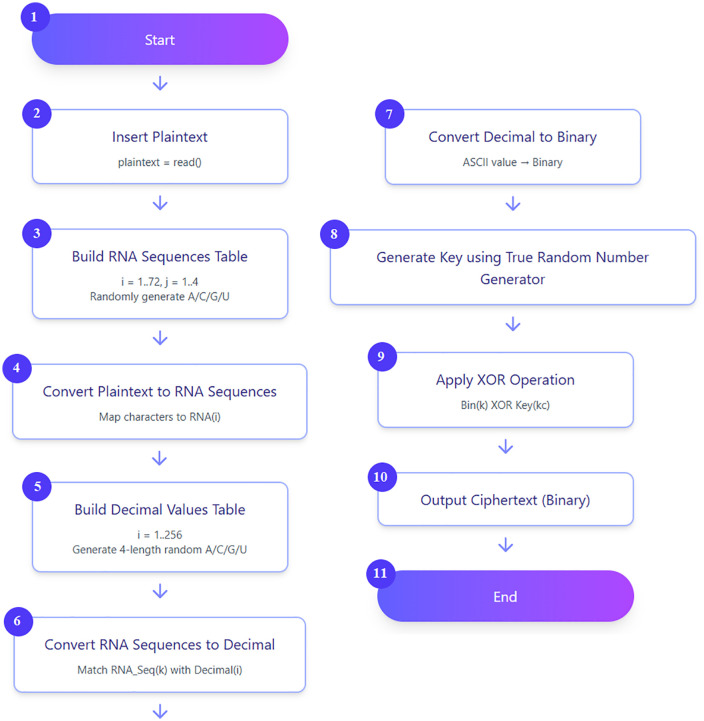
The flowchart of the proposed method.

The third cryptography step is converting the decimal numbers achieved from the previous step to binary format by dividing these decimal numbers by two and noting the remainder until you get to zero. After that, used true random number generator method was used to generate a random and non-predictable key, where this method can play a major role in cryptographic systems because it is widely used in cryptographic applications such as key generation, random padding bits, and authentication protocols. The generated key is used for the next step, where this key must be processed before being used; this is done by converting the generated key to ASCII values and then to binary values. In the last cryptography step, an exclusive or (XOR) operation is applied between the achieved binary values and the generated key. The final binary result obtained by the XOR operation is considered as the ciphertext that will used in the next phase of secure transmission. The steps of the proposed cryptography method are illustrated in Fig 2.

**Fig 3:**
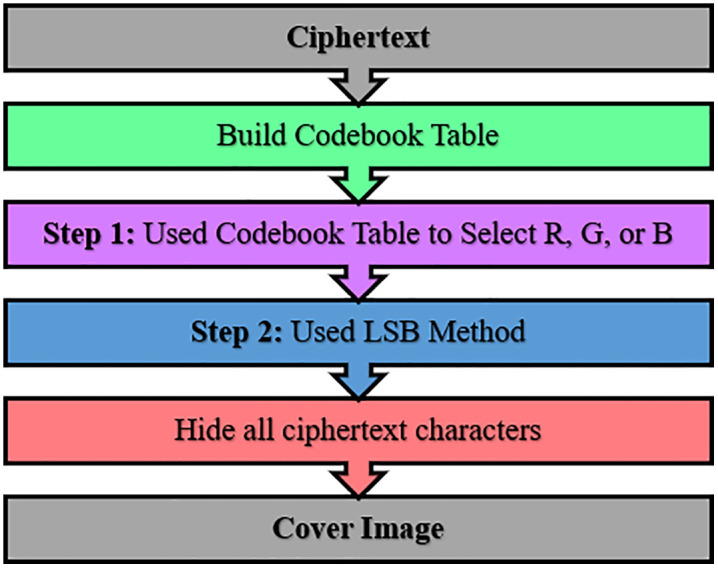
Proposed steganography method steps.

The second phase of the proposed method is steganography, where the main idea of this phase is to hide the ciphertext that is achieved by the first phase into the cover image. This method is done by applying the codebook idea and least significant bit (LSB), where the steps of steganography are shown in Fig 3.

**Fig 4:**
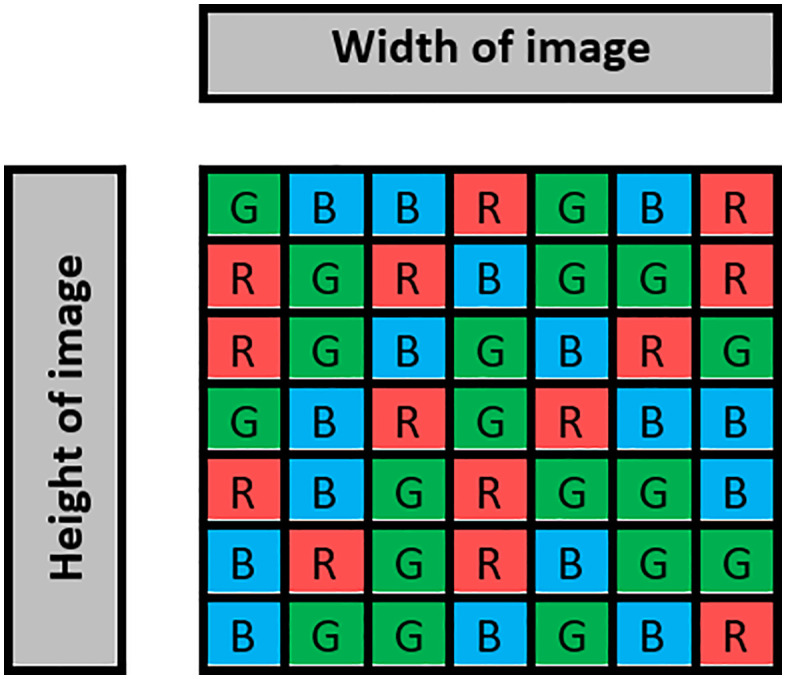
Codebook table.

In the first step, a codebook table is built to select which part of the pixel to store the message inside it (a component of colour), either in red (R), green (G), or blue (B), where the size of the table depends on images size that used to hide a message inside it, if the image has a size of 1024*1024 then codebook size is also 1024*1024. The codebook table is shown in Fig 4. For example, if you randomly select a pixel number four in an image to store a bit inside it, check the same value (four) in the codebook table to select where this bit is stored, either R, G, or B. The codebook plays a crucial role in the proposed steganography step, where the embedding sites of the colours in the RGB channels are determined. The codebook entries are assigned a pixel index and use one of three colour components (R, G or B) through a uniformly random map. This mapping allows making sure that even in case an attacker realizes that LSBs are being embedded, the probability of the distribution of altered bits will be arbitrary. That the codebook is an image of the same size as the cover image and its creation through randomising its generation every time it is transmitted supports resistance to pattern-based steganalysis.

The second step is using the LSB method to hide the bit within the cover image, where this method is used to hide the message without noticeably affecting the image’s appearance. After finishing the steganography process, this image is sent to a specific person (receiver), which includes a secret message inside it.

When the receiver gets the image, two phases to obtain the sending message from this image; these phases are extract and decryption message. In the first phase, used codebook table generated by the sender was used to extract a bit from components of colour, either R, G, or B; this led to extracted all bits from the image, which includes binary values, either 0’s or 1’s. In the second phase, the decryption of the message is applied to get the main message by using the same number of steps in the encryption phase but in reverse order. Where the decryption process is achieved by:

- Receive the image that the sender sends.
- Used the codebook table to decide the place of hidden bits.
- Extract all the hidden bits inside the image.
- Implement XOR operation between extracted binary values and the key value.
- Group and convert each 8 binary number to decimal. For example, the binary number 10000011 is converted to 131.
- Used a decimal converting table to convert each decimal number to four RNA characters.
- Finally, the RNA sequences table is used to convert each four RNA characters to their equivalent characters from the table.
- The achieved characters (alphabet characters, numbers, and special characters) represent the original message sent by the sender.

## 3. Results and discussion

**Fig 5:**
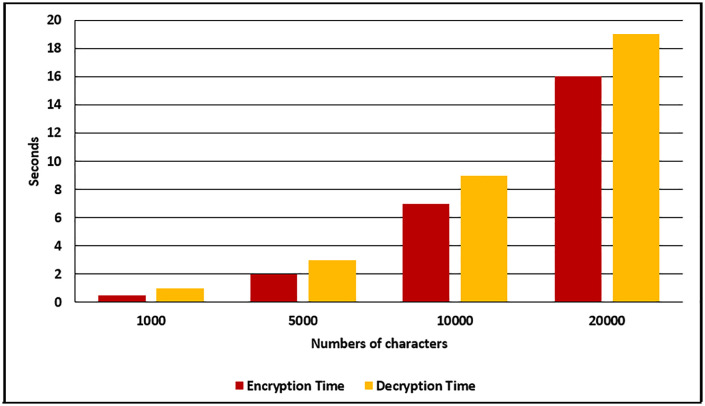
Encryption and decryption time.

The performance evaluation of the proposed method is done by calculating the cryptography process time and steganography process time, where the cryptography process includes encryption and decryption time, while the steganography process includes message hiding and message extraction time. Firstly, the achieved cryptography process time for the proposed method in terms of seconds are shown in Table 3 and Fig 5.

**Table 3:** The achieved encryption and decryption time in terms of seconds.

Numbers of Characters	Encryption Time (s)	Decryption Time (s)
1000	0.5	1
5000	2	3
10000	7	9
20000	16	19

As mentioned in Table 3 and Fig 5, the achieved time of the proposed cryptography method is fast, where the encryption time for different messages with different lengths (1000, 5000, 10000, and 2000 characters) is equal to 0.5, 2, 7, and 16 seconds respectively. At the same time, the decryption time for the same messages is equal to 1, 3, 9, and 19 seconds respectively.

**Fig 6:**
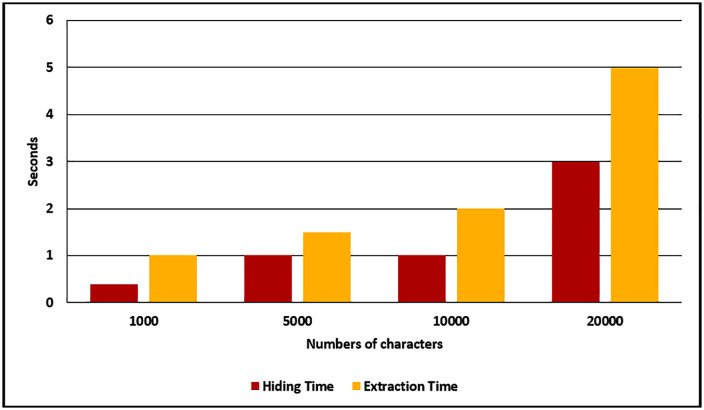
Message hiding and extraction time.

Secondly, the achieved steganography process time for the proposed method in terms of seconds are stated in Table 4 and Fig 6.

**Table 4:** The achieved message hiding and message extraction time in terms of seconds.

Numbers of Characters	Hiding Time (s)	Extraction Time (s)
1000	0.4	1
5000	1	1.5
10000	1	2
20000	3	5

Table 4 and Fig 6 showed that the achieved time of the proposed steganography is fast, where the message hiding time for different message lengths (1000, 5000, 10000, and 2000 characters) are equal to 0.4, 1, 1 and 3 seconds respectively. At the same time, the message extraction time for messages is equal to 1, 1.5, 2 and 5 seconds respectively.

The following example explains and highlights the proposed cryptography process steps; assume that the used plaintext is “Please meet me at street number 62 today at 8 Pm”

The first step of the proposed cryptography method is done by converting the message to RNA Sequences:

GAAG UCAC UAAG AUCU GCUU UAAG AAAA AUGU UAAG UAAG GGUC AAAA AUGU UAAG AAAA AUCU GGUC AAAA GCUU GGUC ACGU UAAG UAAG GGUC AAAA UCGA CCGA AUGU GUAA UAAG ACGU AAAA GAGG UCCA AAAA GGUC GCUA AGGG AUCU AGCU AAAA AUCU GGUC AAAA UUGC AAAA GAAG AUGU

The second step of the proposed cryptography method is done by converting RNA sequences to decimal values:

16 31 27 45 31 136 39 31 31 46 136 39 31 136 27 46 136 45 46 44 31 31 46 136 40 47 39 28 31 44 136 59 55 136 46 41 30 27 51 136 27 46 136 61 136 16 39

In the third step of the proposed cryptography method, decimal values are converted to binary numbers:

00010000 00000000 00011111 00011011 00101101 00011111 10001000 00100111 00011111 00011111 00101110 10001000 00100111 00011111 10001000 00011011 00101110 10001000 00101101 00101110 00101100 00011111 00011111 00101110 10001000 00101000 00101111 00100111 00011100 00011111 00101100 10001000 00111011 00110111 10001000 00101110 00101001 00011110 00011011 00110011 10001000 00011011 00101110 10001000 00111101 10001000 00010000 00100111

While in the fourth step of the proposed cryptography method, a cryptography key is generated, and suppose the generated key is “Cryptography Key” This key is converted to decimal values and then converted to binary values:

67 114 121 112 116 111 103 114 97 112 104 121 32 75 101 121

01000011 01110010 01111001 01110000 01110100 01101111 01100111 01110010 01100001 01110000 01101000 01111001 00100000 01001011 01100101 01111001

The last step of the proposed cryptography method is done by applying the XOR operation between binary values achieved from step 3 (the message) and binary values achieved from step 4 (the key). The achieved results represent the ciphertext:

01010011 01110010 01100110 01101011 01011001 01110000 11101111 01010101 01111110 01101111 01000110 11110001 00000111 01010100 11101101 01100010 01101101 11111010 01010100 01011110 01011000 01110000 01111000 01011100 11101001 01011000 01000111 01011110 00111100 01010100 01001001 11110001 01111000 01000101 11110001 01011110 01011101 01110001 01111100 01000001 11101001 01101011 01000110 11110001 00011101 11000011 01110101 01011110

## 4. Conclusion

This study proposes a hybrid approach to secure text transmission based on cryptography and steganography as a more secure and reliable approach. The proposed cryptography depends on the benefits of using RNA, where the sending message is firstly converted to RNA sequences by building an RNA sequence generation table. These sequences are converted to decimal values, then convert these values to binary format. After that, True Random Number Generators are used to generate the encoding key, and finally, XOR operations are applied to achieve the ciphertext. When the cryptography process is complete. The obtained binary (ciphertext) is hidden within an image by building a codebook table to decide on colour components to store the bit inside it and by using the LSB method to hide the same bit without affecting the image. The achieved results showed that the proposed system provides a secure platform for sharing secret information through fast time. For future work, the improvements for the proposed work can be done by using deep learning based key generators, guided generation of codebooks by image features and lightweight cryptographic transformations to enable application in resource-limited devices such as IoT sensors.
